# The Status of Wildlife in Protected Areas Compared to Non-Protected Areas of Kenya

**DOI:** 10.1371/journal.pone.0006140

**Published:** 2009-07-08

**Authors:** David Western, Samantha Russell, Innes Cuthill

**Affiliations:** 1 African Conservation Centre, Nairobi, Kenya; 2 School of Biological Sciences, Bristol University, Bristol, United Kingdom; University of Pretoria, South Africa

## Abstract

We compile over 270 wildlife counts of Kenya's wildlife populations conducted over the last 30 years to compare trends in national parks and reserves with adjacent ecosystems and country-wide trends. The study shows the importance of discriminating human-induced changes from natural population oscillations related to rainfall and ecological factors. National park and reserve populations have declined sharply over the last 30 years, at a rate similar to non-protected areas and country-wide trends. The protected area losses reflect in part their poor coverage of seasonal ungulate migrations. The losses vary among parks. The largest parks, Tsavo East, Tsavo West and Meru, account for a disproportionate share of the losses due to habitat change and the difficulty of protecting large remote parks. The losses in Kenya's parks add to growing evidence for wildlife declines inside as well as outside African parks. The losses point to the need to quantify the performance of conservation policies and promote integrated landscape practices that combine parks with private and community-based measures.

## Introduction

The need for ecosystem-wide monitoring has become more pressing as the goals of conservation have expanded from saving endangered species and national parks to sustaining biological diversity, ecosystem function and ecological services [1], [2], [3]. Quantification of species trends and the factors governing population and ecosystem viability are vital to forecasting, planning and managing wildlife populations, and in auditing the success of alternative conservation policies and practices.

Despite the need to quantify conservation programs, few studies have looked at the success of protected areas, which now cover 10% of the earth's land surface [4], relative to non-protected areas [5]. Several factors account for the paucity of conservation audits. First, the level of monitoring needed to assess conservation performance is expensive and calls for long-term commitment and planning. Research priorities have focused on charismatic species and the most urgent conservation threats. Long-term ecological monitoring has, consequently, been given little attention [6], [7] until the establishment of a network of Long Term Ecological Research sites [8]. Exceptions for large mammal ecosystems include long-term ungulate counts in Africa, conducted in national parks such as Kruger [9], Serengeti [10], Ngorongoro [11] Maasai Mara [12] Nairobi [13], [14] and Nakuru [15]. These counts provide population trends for individual parks, but do not compare the success of parks per se with similar non-protected areas, or the protected area systems as a whole with country-wide wildlife trends. Second, there has been little coordination among individual researchers, conservation organizations, government agencies or landowners conducting wildlife censuses. The lack of coordination and standardization creates methodological problems in comparing discontinuous data and different counting methods [5]. Data are often hard to locate, verify and synthesize because they are so scattered in agency reports, private files and journals. Third, complex ecological interactions such as rainfall-ungulate and predator-prey oscillations make it difficult to distinguish human-induced from background ecological changes. Owen-Smith and Ogutu [16] underscore the importance of long-term systematic monitoring in Kruger National Park for teasing out the impact of conservation policies and management practises from rainfall, predation and other ecological factors.

Lamenting the lack of quantitative data, Struhsaker et al. [17] used questionnaire surveys to gauge the success of protected areas relative to community-based conservation and non-protected areas in Africa. Questionnaires are, however, subjective and may aggravate rather than resolve debates over conservation policies and paradigms [18], [19]. Sutherland et al. [20] noted that conservation practice relies more on anecdote and myth than quantitative evidence and called for more evidence-based conservation.

Despite a lack of systematic monitoring, there has been a large number of individual wildlife censuses conducted in eastern and southern Africa since the 1960s. Scholte and Caro [5] have shown that it is possible to statistically combine such disparate counts and methodologies to compare protected area with non-protected areas systems. To compare wildlife trends as a function of protected area status in Tanzania, Scholte and Caro [5] compiled censuses for seven census zones over two time periods a decade apart (late 1980s-early 1990s with late 1990s-early 2000s). The aggregate population trends show wildlife declining in all census zones over the decade, but with the level of protection significantly slowing declines and in some species reversing trends.

Caro and Scholte [5] point out that a raft of studies now point to ungulate declines inside as well as outside parks across Africa. If substantiated, the declines raise grave concerns about the adequacy of parks and point to the need for a radical review of conservation policies. A major review should, however, be grounded in more substantial evidence about the park trends and the underlying causes. Deficiencies in boundary design and area coverage or inadequate protection and ecological management [21], [22] could account for the losses. The first calls for major changes in national conservation policy, the second for changes in parks' management practices. Quantifying the importance of parks in conserving wildlife, as well as quantifying the wildlife trends and their causes, calls for a serious investment in ecological monitoring. The monitoring should include multi-species censuses and environmental variables in order to tease out human-induced from natural trends, and to provide a quantitative audit and comparative analysis of conservation strategies.

Here we assemble continuous multi-species ungulate censuses of sufficient duration and on a large enough scale to transcend climatic cycles and to compare protected areas with matching non-protected areas of Kenya. We also compare the importance of Kenya's protected area system relative to country-wide wildlife numbers and trends.

Wildlife audits of the rangelands have been conducted by the government's Department of Remote Sensing and Resource Surveys (DRSRS) since 1977. The rangelands cover three quarters of Kenya's 440,000 km^2^ land surface and all but a small proportion of its large herbivore populations [23], [24]. The counts cover all species Thomson's gazelle-sized (15 kg) and larger, giving a good measure of the large ungulate community which dominates the savannas [25]. The DRSRS national audits show that wildlife has declined by more than a third over the last 25 years [23], [24]. Due to the uncoordinated nature of counts and scattered results, no such audit of national parks and reserves has been conducted, despite counts dating from as early as the 1950s and 1960s [13], [26].

Here we assemble over 270 counts conducted over the last 25 years or more to assess wildlife trends in national parks relative to countrywide trends. The counts include published censuses and formal reports where possible, but most are drawn from unpublished counts from public institutions, individual researchers and volunteer groups.

Kenya has 23 terrestrial national parks under the administration of the Kenya Wildlife Service and 26 national reserves under district administration. Collectively, the parks and reserves cover 8% of the national land surface of Kenya. Many parks and reserves have too few counts to assess long-term trends. We have therefore included in our study all parks that had a baseline count by 1977 and have been counted repeatedly until at least 1997, giving 20 years of contemporaneous data.

The study includes 73% of the area covered by national parks and an estimated 95% of the national wildlife population [23]. Unfortunately, data is only available for one national reserve, Maasai Mara, which is under district administration. The Maasai Mara does, however, account for most of the wildlife found in national reserves. Grunblatt et al. [23], calculate that the remaining national reserves account for 32% of all national protected area coverage in Kenya, but only 2% of the national wildlife population. The sparse populations in national reserves reflect their marginal wildlife importance in most cases, as well as heavy livestock occupation and poor protection.

Our audit of Kenya's protected areas was analyzed using standard methodologies with four objectives in mind. First, we assess wildlife numbers and trends in one of Africa's premier protected area systems. Second, we compare trends in protected and non-protected areas similar in setting. We did so by matching contemporaneous counts inside and outside the park within the same ecosystem. Third, we compare wildlife trends in parks with nation-wide trends. Fourth, we compare the wildlife coverage given by protected areas as a proportion of national totals. We look at the numbers of all species combined rather than individual species in order to compare the trends and overall contribution of wildlife in parks to national trends and to the country-wide population. A more detailed study underway looks at species trends and changes in guild and community structure.

## Results

### Trends in National Parks and Reserves

Linear regression models were fitted using the Prais-Winsten Generalised Least Squares method, assuming errors have a first-order autocorrelation structure. The assumption of first-order autoregression was verified by partial autocorrelation of the raw data. Analyses were performed using SPSS 12.0 for Windows. All values were log10 transformed prior to analysis. Data for any missing years were estimated by linear interpolation.

Highly significant declines have occurred in three of the seven parks. These include Tsavo East and Tsavo West National Parks (combined) and Meru National Park. Nairobi National Park shows a negative but non-significant downward trend. Mara also shows a negative but insignificant decline. However, an earlier study [12], based on more complete censuses than we were able to obtain, concluded that non-migratory wildlife in Mara National Reserve declined by 58% between 1977 and 1997, and that there was no significant difference in declines in and outside the reserve [12]. Nakuru and Amboseli show non-significant increases. The five protected areas showing declines are Kenya's most populous wildlife preserves. Collectively, these parks account for 98% of wildlife covered by the protected areas listed in Table 1. The largest parks show the steepest declines. Wildlife populations declined 63% in Tsavo East and West between 1977 and 1997 and 78% in Meru between 1977 and 2000. There are, furthermore, indications that wildlife populations in the smaller parks have declined in more recent years as shown in Table 2 below.

**Table 1:** Trends in large mammal numbers for key parks, reserves and adjoining non-protected areas within the ecosystem.

	Slope (b)	T	P value	Trend and significance	Count period	N
Tsavo NP	−0.017	4.53	0.0003	− ****	1977–1997	11
Tsavo Outside	−0.030	4.76	0.0002	− ****	1977–1997	11
Mara NR	−0.008	0.53	0.6006	**−**	1977–1997	21
Mara Outside	−0.020	2.77	0.0125	− **	1977–1997	21
Amboseli NP	0.001	0.21	0.8323	+	1969–2005	44
Amboseli Outside	0.005	0.68	0.5001	+	1973–2005	32
Kitengela	−0.010	1.16	0.2574	−	1977–2002	17
Nairobi NP	−0.000	0.07	0.9418	−	1961–2002	30
Nakuru NP	0.007	0.62	0.5413	+	1970–2002	23
Meru NP	−0.029	10.82	<0.00001	− ****	1977–2000	3

The table includes the number of counts for each area (N). Significance values are P<0.1 (*), P<0.05 (**), P<0.01 (***) and P<0.001(****).

**Table 2:** Trends in large mammal populations in the three smallest National Parks of the study from 1990 onwards.

Park	Slope (b)	T	P	Period of data	Trend and Significance
Nairobi	−0.034	2.308	0.044	1990–2002	−**
Nakuru	−0.014	2.918	0.015	1990–2002	− **
Amboseli	−0.049	13.655	0.000	1990–2002	− ****

**Figure 1:**
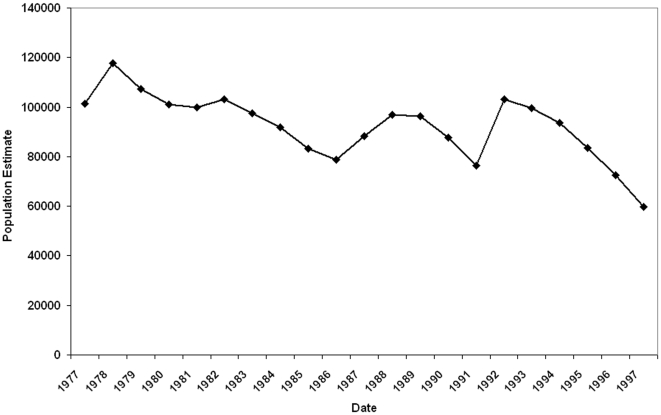
Combined wildlife population changes for Tsavo East, Tsavo West, Amboseli, Nakuru, Meru and Nairobi National Parks and between 1977 and 1997.

The combined wildlife population change for all national parks listed in Table 1 is given in Figure 1 for the period 1977 to 1997. The data include interpolated counts for Tsavo East and West, Amboseli, Nakuru, Nairobi and Meru. The decline is highly significant (b = −0.008, t = −3.066, p = 0.007). The overall percentage loss of wildlife for all five parks is 41%. The percentage loss for Maasai Mara National Reserve over the same period was 25%.

### Trends in Protected Areas and Adjacent Ecosystems

A comparison of wildlife trends in nationally protected areas and adjacent ecosystems is given in Table 1. Table 3 gives the values for the interaction term, which formally tests for a significant difference in the slopes (log10 numbers regressed against Year) inside and outside a given park. Analyses were performed using S-Plus. No interactions are significant, showing that yearly changes do not differ significantly inside and outside parks in the four matching areas for which data are available. No such data are available for Meru National Park. However, data for the adjacent districts of Isiolo and Samburu [24] suggest the trend outside is also steeply downwards. In the case of Nakuru, the park is ecologically isolated from the surrounding farms by an electric fence, so has no matching ecosystem.

**Table 3:** The magnitudes and significance of interactions between yearly changes within parks and adjacent ecosystems.

	Slope(b)	Se(b)	T	P
Tsavo	−0.01142	0.0116	0.98455	0.3311
Mara	−0.01133	0.02087	0.54313	0.5902
Amboseli	−0.02191	0.02686	0.81573	0.4197
Nairobi/Kitengela	−0.043	0.0358	1.19994	0.2376

**Figure 2:**
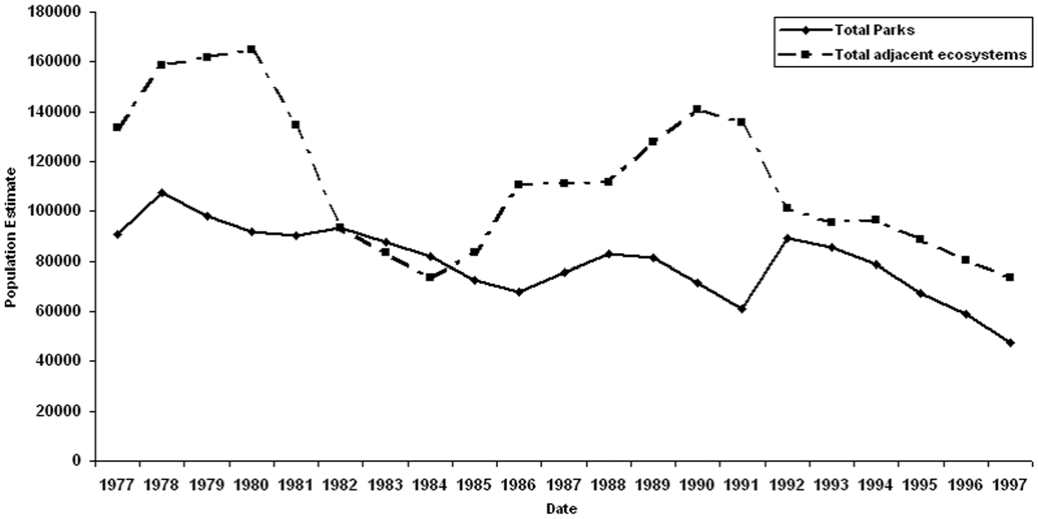
Total wildlife populations for national parks with matching external ecosystem counts. The parks include Tsavo East, Tsavo West, Amboseli, Nairobi but exclude Meru and Nakuru.

In Figure 2 we summarize wildlife numbers for nationally protected areas with matching ecosystems for the period 1977 to 1997, the maximum period for which there are contemporaneous counts for all areas. We have excluded Maasai Mara from this analysis because we were unable to get the full set of counts and because of the large distortion a seasonal influx of migratory wildebeest from Serengeti in Tanzania has on the resident totals for Kenya's protected areas [23].

The combined wildlife populations show considerable fluctuation in parks and adjoining areas, with numbers rising in the late 1970s, falling through to the mid-1980s, rising again more slowly in the late 1980s and falling steeply in the 1990s. The large fluctuations outside protected areas is likely due to their greater proportion of wet season range than parks and their more episodic use, especially with increasing settlement [27].

The fluctuations of populations outside and inside parks are closely correlated (r = 0.51, p = 0.0164) and not significantly different in slope (b = 0.00081, standard error = 0.0126, t = 0.0638, p = 0.9495). Although it is not possible to relate the national wildlife trends to rainfall, the oscillations correspond to drought cycles recorded for southern Kenya [28], where the majority of wildlife is located. Independent evidence for fluctuations due to drought and rainfall fluxes has been shown for Nairobi [29], Tsavo [30] Amboseli [31] and Maasai Mara [12]. Climatically linked ungulate fluxes are to be expected, given the close correlation between large herbivore biomass and rainfall across a wide range of savanna ecosystems in eastern and southern Africa [32], [33], [34].

Despite the large inter-annual populations, the counts show a steep decline in wildlife populations in parks and adjacent ecosystems transcending drought cycles. The decline in parks is highly significant (b = −0.011, t = −3.773, p = <0.001). Aggregated wildlife populations in parks declined by 48%, from 90,691 to 47,599 between 1977 and 1997. Adjoining area populations declined by 45%, from 133,758 in 1977 to 73,394 in 1997.

### Protected Areas and National Audits Compared

A meta-analysis of the DRSRS censuses of the Kenya rangelands counts between 1977 and 1997 showed a highly significantly decline in numbers [24]. Wildlife estimates derived from the regression equations for 17 districts' censuses show a nationwide decline of 38% in wildlife numbers. Based on the data in Figure 1, wildlife populations for the combined national parks show a loss of 41% over the same period. Grunblatt et al. [23] earlier showed a loss of 32% of wildlife in Kenya rangelands between 1977 and 1994. The similar losses inside and outside protected areas as a whole reflect the losses for parks and matching ecosystems (Table 3). The parallel trends show that parks and reserves have not insulated wildlife from the steep country-wide declines of the last 30 years.

The importance of Kenya's protected areas can be gauged by comparing the proportion of wildlife found in parks and reserves with the national total (Table 4).

**Table 4:** Percentages of wildlife found within protected areas relative to national totals, averaged for the 1990's.

Conservation Status	Wildlife totals	Percent of all wildlife
National Parks	82,957	10
Maasai Mara National Reserve	208,405	25
All Nationally Protected Areas	291,363	35
Total National Population	846,652	100

Based on the national audit for the 1990s, national parks account for approximately 10% of all Kenya's wildlife and national parks and national reserves for 35%. Maasai Mara accounts for 25% of the national total, underscoring its singular importance in Kenya's protected area system.

## Discussion

Our results have specific and general implications for conservation. Specifically, the decline in Kenya's park populations is not surprising, given the inherent shortcomings in their design. Only a modest portion of the annual migratory range of large herbivores is included in Kenya's parks. Most parks differentially cover dry season rather than wet season ranges of the dominant migratory species such as wildebeest and zebra [27]. Seasonal range losses will therefore reduce parks' populations too [35].

Big parks in Kenya are no more insulated from the wildlife decline than small parks. The three largest protected areas, Tsavo (East and West), Meru and Maasai Mara [12], have the steepest wildlife losses. Poaching may account for a significant portion of the losses in Meru, but is unlikely to account for much of the losses in Tsavo or Mara. In general the security provided by the Kenya Wildlife Service since 1989 has contained poaching, as evidenced by the steady increase in rhinos [36] and elephants [37], the two species most vulnerable to poaching. Range loss in the herbivore migratory areas has been shown to account for most of the population losses in Mara [35]. Range loss due to agricultural expansion may also account for a portion of the losses in Tsavo. Habitat change and segregation effects caused by the spatial segregation of previously interlinked movements of wildlife and pastoralists in the savannas are also likely candidate causes [38], [39]. It will take refined research to decipher the relative weighting of such causes.

Two of the smallest parks, Nakuru and Amboseli, showed non-significant upward trends in population between 1977 and 1997 (Table 1), but significant declines since 1990 (Table 2). The upward trend in both cases is explained by the exclusion of livestock after the creation of the parks and compensatory increase in wildlife, the downward trend by the dry conditions prevailing between the 1990s and 2000s [28]. In the case of Amboseli, the engagement of communities around the park in tourism revenues was also a strong contributing factor to the wildlife increase [31].

More generally, long-term monitoring in Kenya adds to growing evidence of wildlife declines in many African parks [5]. For example, Scholte et al [40] highlight the severe decline of a number of species of antelope in the Waza National Park in Cameroon over the last 40 years, due to interacting effects of changes in rainfall, flooding and human interventions. In the Kruger National Park in South Africa, roan antelope have declined from about 450 to 45 individuals between 1986 and 1993 [41], matched by similar declines in sable and tsessebe [42]. The total of all non-migratory wildlife species in the Maasai Mara ecosystem has declined by 58% in the last 20 years [12]. Ngorongoro Crater has experienced a decline in wildebeest, Grant's and Thompson's gazelles since the mid-1980s [11].

The evidence of park losses points to the need for systematic monitoring of ecological trends and biological criteria for auditing conservation policies and practices. The results show sufficient variation in conservation areas and approaches to begin weighing the relative importance of various policies, strategies and management practices in conservation [41], [43], [44], [11]. Evidence from Tanzanian parks, for example, suggests a better track record than Kenya [44]. The high caliber of Kenya's security services rules out poaching as a factor. Two plausible additive hypotheses are, first, the larger size and greater ecological integrity of Tanzanian parks relative to Kenya's and, second, Kenya's lack of habitat management, especially rangeland burning, to counter pasture maturation and segregation effects [45], [38].

The value of large-scale long-term trend analysis is highlighted in a recent study showing that wildlife on private and community sanctuaries is stable or increasing [46], in contrast to the declines in protected areas and country-wide. The results of this study and our own findings suggest that parks associated with community and private conservation initiatives do better than parks with no outreach programs. Such evidence points to the need for new policies that combine national, private and community initiatives in order to sustain large free-ranging herbivore populations at an ecosystem and landscape scale [27].

## Materials and Methods

The count data were obtained from the Department of Remote Sensing and Resource Surveys (DRSRS) for Tsavo East and West and the Kitengela, from DRSRS and Ottichilo [35] for Maasai Mara, from the Kenya Wildlife Service for Nairobi National Park and Nakuru, from Kenya Wildlife Service and Ian Douglas-Hamilton and Hillman [47] for Meru National Park and the Amboseli Research and Conservation Project for Amboseli [31].

Species covered by the surveys include: elephant (*Loxidonta Africana*), buffalo (*Syncerus caffer*), Burchell's zebra (*Equus burchelli*), giraffe (*Giraffa camelopardalis* ), wildebeest (*Connochaetes taurinus*), eland (*Taurotragus oryx*), waterbuck (*Kobus allipsiprymnus* ), warthog (*Phacochoerus africanus*), Grant's gazelle (*Gazelle granti*), Thomson's gazelle (*Gazelle thomsonii*), impala (*Aepyceros melampus*), lesser kudu (*Tragelaphus imberbis*) oryx (*Oryx gazella*), black rhinoceros (*Diceros bicornis*), topi (*Damaliscus korigum*) and hartebeest (*Alcelaphus buselaphus*),
